# Music, families and interaction (MUFASA): a protocol article for an RCT study

**DOI:** 10.1186/s40359-022-00957-8

**Published:** 2022-11-04

**Authors:** Stine L. Jacobsen, Gustavo Gattino, Ulla Holck, Julie Ørnholt Bøtker

**Affiliations:** Department of Communication and Psychology, Aalborg University, Aalborg, Denmark

**Keywords:** Music therapy, Community music, Families, Interaction, Prevention

## Abstract

**Background:**

General mental health and interpersonal skills of families are crucial to children's development and future. Research suggests a link between participation in music activities and individuals' own sense of mental health, as well as an effect in objective measures of health such as quality of life, social skills, and rehabilitation of various kinds. However, in Denmark there are not many services for families with school-aged children (7–12 years) that focus on prevention and strengthening family interaction hereby minimising the risk of children not thriving and developing optimally in terms of social and emotional skills and competences. The purpose of this study is to investigate the effect of shared music activities on family interaction, parental stress, and child well-being.

**Methods:**

The study is a controlled effect study where 40 family dyads from Aalborg and Aarhus are randomized into 4 different groups including music therapy activities, community music, family concerts and a control group. Measurements of family interaction (Assessment of Parent Child Interaction, APCI) and mental health (Parental Stress Scale, PSS, and the Strengths and Difficulties Questionnaire, SDQ) will be made at baseline/pre intervention, at post intervention and a follow-up measurement 12 month after baseline (month 1, 3 and 12) 3 times in total. Furthermore, data about the family dyad’s regular participation in music events as part of every-day life at the same measure points (month 1, 3 and 12). Since this is a planned prospective study, results are not yet available, but clinical experience from a feasibility study in 2021 were promising and expected challenges and changes are discussed in the article.

**Discussion:**

Findings of this study will be relevant for all fields where music is applied for families, such as education, mental health, social work and for basic research on the study of music and interaction. Music used as a resource for families is crucial in understanding how different modalities can influence the family interaction including emotional communication and attachment.

*Trial registration*: ISRCTN, ISRCTN17290015, Registered 03 March 2022, https://www.isrctn.com/ISRCTN17290015

**Date and protocol version:**

July 2022, version 1. Protocol is planned to be updated after finalized recruitment during second data collection point and again after the third and last data collection point (see Additional file 1: SPIRIT Checklist).

**Supplementary Information:**

The online version contains supplementary material available at 10.1186/s40359-022-00957-8.

## Background

World Health Organization reports that at least 55 million children in the European Region are affected by child maltreatment. Child neglect or child maltreatment has great human costs and is serious societal challenge as it risks healthy child development [1]. Several studies and government reports urge us to develop better prevention strategies designed to reduce risk factors and enhance protective factors by promoting factors for optimal family functioning [1–3] We must organise varied prevention interventions for children, young people, and their parents throughout the school years, with the aim of strengthening family interaction. Research studies on the impact of the current pandemic on family functioning indicate a further need for widespread family support and intervention to prevent and reduce parent and child mental health and behavior problems during crises recommending promoting individual and family resilience [4]. Thus, there is a need for more resource-focused services for all families with school-age children, and specifically more knowledge about effective long-term interventions.

We know from several studies that there is a clear link between participation in music activities and individuals' own sense of mental health, as well as effect on objective measures of health such as quality of life, social skills, and rehabilitation of various kinds [5–8]. Research indicates that doing music together creates joy and a sense of connection between those who play or sing as this supports the ability to listen, tune and synchronise with each other all of which are basic elements of all healthy relationships [9, 10]. A recent study on parent–child musical engagement in childhood and adolescence as a predictor of relational quality in emerging adulthood concluded that joint musical engagement is associated with stronger interpersonal relationships in the family [11]. Furthermore, recent music therapy research and literature tells us that through music therapy, vulnerable children, and families with different types of problems can improve their communication and increase well-being and health including improved attachment, engagement, interaction, responsiveness and sensitivity between parent and child [12–18]. Music therapy with families can also positively influence children and parents individually including developing better social, emotional, and cognitive skills in the child as well as reducing stress, anxiety and depression in different levels and stages and creating better well-being in the parent [19, 20].

Research on community music specifically with families is sparse, but there is research on various other community music activities. Recent research is gradually focusing less on cognitive and reasoning skills and more on emotional and social skills when examining the effects of community music [21, 22]. Emotional training is increasingly understood as an innovative educational tool that is useful in addressing the many social and general education needs and challenges. There is some scientific evidence on the positive effects of good social and emotional skills including, among others, increased self-esteem, learning enhancement and motivation, good adjustment, and participation in school, and better relationships with peers and teachers [23, 24]. Other studies examine the effects of community music, where it becomes clear that social and emotional skills are improved in participants who are actively involved in making music with others [21, 25, 26]. Research often builds on an understanding that active music-sharing naturally requires or demands collaborative skills and a desire to work together including respect for others, the importance of helping others, and a focus on redressing inequalities [27, 28].

The mechanisms of family communication can be understood on the perspective of developmental psychology and complex theories of early non-verbal parent–child interactions. Interpersonal interaction relies mainly on nonverbal forms of communication. Verbal language is not enough to express the quality, intensity, and different nuances of our emotions [29]. The ability to send, receive and decode non-verbal messages is an important part of our ability to communicate—both verbally and non-verbally [30]. According to Malloch and Trevarthen, we all fundamentally possess a communicative musicality that we use when we learn to communicate nonverbally and learn to understand emotion and express emotion [31]. We learn through playing with sounds and the theory of communicative musicality provides a framework for understanding how play occurs naturally and spontaneously in musical interactions [31]. Communicative musicality and non-verbal communication are essential when it comes to building and improving interaction patterns and communication skills in the family. Musical interaction can be offered as a potential route to repairing difficulties in early and later parent-infant interaction. Using music enables the family to try out and explore different communication patterns aiming to break inappropriate patterns, and here non-verbal communication skills are thus essential in the development of self-regulation. Supporting the child to become emotionally stable and emotionally self-regulated is one of the key aspects of parenting and parenting skills [32]. Stern further describes how intersubjective experiences and affect attunement between parent and child are necessary for the child to develop a robust emotional self [33]. For example, through exploration, play, and interaction with non-verbal sounds, the infant learns about complex behaviours and can gradually recognise and understand some behavioural elements and patterns better than others. Attachment theories are often associated with affective attunement, as the quality of attachment between parent and child depends on the emotional availability, responsiveness, and sensitivity of the parent over time. Music activities are often described as a positive approach that enables ongoing work on attachment, as meaningful moments can occur despite the most severe and uncertain circumstances [20].

Despite our knowledge from international research studies that music experiences of various kind can have a positive effect for many different types of families, too few music-based and resource-focused activities are offered to families with school aged children. To aid society challenges and prevent child neglect and child maltreatment, we need more knowledge about how music activities can be used more consciously and if it can be used to strengthen family life.

## Objectives

The objectives of this study are as follows:

- To investigate how targeted music activities can strengthen family interaction including music therapy activities, community music activities and family music concerts.
- To investigate if different formats of music experiences can strengthen families including their interactions well as individual measures of well-being and parental stress.

It is predicted that through the targeted use of music therapy methods, group music therapy can enable families to strengthen family interaction as well as improve general well-being, decrease levels of stress in the parent and enhance the well-being of the child. Also, it is expected that through targeted community music activities, community group music can provide families with opportunities to strengthen family interactions as well as general well-being and decreased levels of stress in the parent and enhance well-being in the child. Finally, it is predicted that participating in family concert will provide the families with opportunities to increase general well-being and decrease levels of stress in the parent and strengthen the well-being of the child.

## Method

### Participants

The study will include voluntary families invited from primary schools in Aalborg and Aarhus municipalities and invited through social media posts on Facebook, school intranets and university website. Dyads of one parent and one child participating in the study will comply to the following inclusion criteria**:** Voluntary families with school-age children aged 7–9 from the cities of Aalborg and Aarhus.

### Study design

**Fig. 1:**
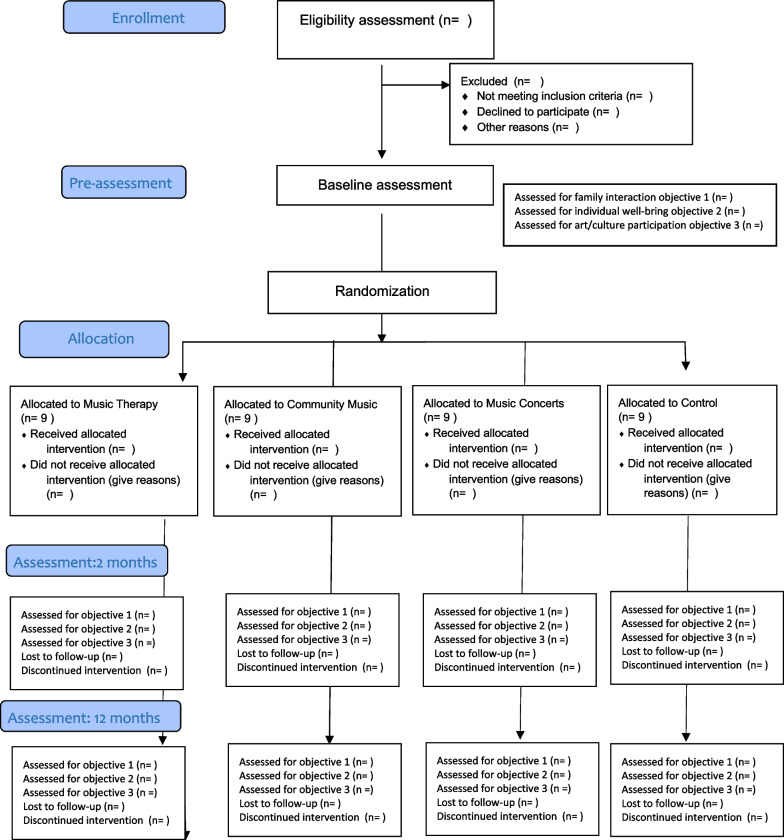
Consort Flowchart

The project is both a classical controlled effect study (pilot, before, after and follow-up measurement) and a correlation study of the relationship between participation in everyday art/music activities and parental stress, child well-being and parent–child interaction in the family. After inclusion in the study and baseline assessment, participants will be assigned to one of the study conditions. Paired samples randomization is implemented to ensure equal distribution of types of families between each intervention and control. The allocation ratio of intended numbers of families in the comparison groups will be 1:1:1 so that the number of families receiving each intervention will be similar. Before random assignment is performed, it has to be confirmed by the investigator recruiting participants that the eligibility criteria have been met and participants are formally enrolled. We decided to have three dyads in the MT group and five in the CM group. Once recruitment and data collection at baseline are complete and informed consent to participate in the study has been obtained, the respective randomisation code will be revealed to the investigator by an administrative person at the central randomisation office who will have no contact to participants. An overview of the study design is shown in Fig. 1

### Interventions

When comparing different interventions, it is important to be able to identify differences between the interventions. Waltz and colleagues [34] discuss how to secure interventions and keep them flexible to meet the need of participants. They suggest a differentiation between four different layers: Unique and and essential, essential, compatible, and proscribed [40]. Working with the creative media of music and the dynamic complexity of families, developing a treatment guide can be quite a challenge. However, several music therapy research projects have successfully addressed the issue of creating clear intervention manuals and treatment guides [35–37]. Below are intervention guides for the active interventions including family-centered music therapy and community music therapy implemented in the MUFASA project including underlying theory, goals, role of the therapist, role of music, forms of intervention, techniques, and methods. The intervention guides are also presented in Table 1 below the descriptions where the differences and similarities are more distinct. The are no specific guidelines for family concerts as they are of more receptive nature and not possible to control or impact within the scope of this project. However, the project did create criteria for which types of family concerts to include which are also described below.

**Table 1:** Treatment guidelines

Levels	Experience-oriented music therapy		Community music	
Essential and unique	Goals	Strengthen awareness of and clear emotional communication and interaction in the family	Goals	Positive and shared musical experience and expressions Explore and increase musical creativity and musical skills
	Role of tp,	Balance role modelling vs facilitating interaction Family-led and family centered Attune to emotional needs of family Be informed by family dynamics and patterns Take active part in musical activities	Role of facilitator,	Making active music-making accessible Facilitator/teacher lead Be informed by and adapt to individual musical/personal skills and group diversity Take active part in musical activites
	Role of music	Reflect family communication and interaction Can change/strengthen/vitalize family interaction Enables sharing of emotions and thoughts Can have emotional/mood content	Role of music	Enables shared aesthetic and vitalizing experiences and expressions Can motivate engagement and involvement
	Activities	Improvisations targeting nonverbal emotional communication Explore and experience own or other family patterns/traits/identity using music Trying out different use of instruments/music	Activities	Structured music activities that enable positive music experience and expression Repetition and rehearsing Music product oriented activities
Essential	Goals	Create safe environment Facilitate positive togetherness	Goals	Create safe environment Strengthen positive family interaction and self-confidence
	Role of tp,	Include all family members Adjust to musical skills and personal traits Accommodating, playful and flexible Facilitate use of instruments Refer on when relevant Choose intervention and activities in relation to own musical and therapeutic competences	Role of facilitator,	Include all family members Accommodating, playful and flexible Facilitate use of instruments Choose intervention and activities in relation to own musical and teaching competences
	Role of music	Can motivate engagement and involvement Enables aesthetic/vitalizing experiences Common third	Role of music	Common third Enables socialization
	Activities	Guided group-based use of music	Activities	Teacher/facilitator guided group-based use of music
Compatible	Goals	Strengthen musical skills and self-confidence Enable new friendship between families Strengthen conscious use of music	Goals	Being aware of musical genres and preferences New friendships and deeper relationships
	Role of tp,	Educating about healthy use of music	Role of facilitator,	Educating about genres Motivating through own musical skills
	Role of music	Reminiscing and explore identity	Role of music	Exploring identity and reminiscing
	Activities	All (other) music therapy related techniques	Activities	All (other) community music techniques
Proscribed	Goals	Increase of stress and anxiety in family members Worsen and confuse family communication and interaction Targeting trauma or potential trauma in family members	Goals	Increase of stress and anxiety in family members Decrease sense of mastery and self-confidence Targeting trauma or potential trauma in family members
	Role of tp,	Exclude, devaluating or judging family members create skewed alliances, over simplify, over-react Superior appearance Working in depth with group or individuals Don’t start what you cannot finish	Role of facilitator,	Exclude, devaluating or judging family members Focus on individuals or individual families, Superior appearance
	Role of music	Over stimulation or overwhelming	Role of music	Over stimulation or overwhelming
	Activities	Activities impossible to master Inappropriate violence or aggression Inappropriate power struggle activities Activities risking harming the instruments beyond repair	Activities	Activities impossible to master Inappropriate violence or aggression Inappropriate power struggle/competition activities Activities risking harming the instruments beyond repair
Theoretical foundation		Music therapy Community music therapy		Community music

### Music therapy intervention

The unique and essential goal is to provide the family with the opportunity to strengthen their emotional communication and interaction. It is about creating a safe, respectful, and non-judgmental environment where the family can experience positive interaction, strengthen their confidence, and where they can play and explore different ways of being together. New friendships with other families might evolve, and the family might become more aware of the use of music (either as individuals and/or as a family) but this is not essential. Instead, is about making the family believe in themselves, strengthening their coping strategies and resilience and making them the primary change agents in their family life. The goal is to help the family to become clearer to each other, to find their own inner resources and to help them find good ways of being together [38].

The approach is mainly resource-oriented with a focus on adapting to the family's individual needs. Working with flexibility and variation can help strengthen the family's ability to meet and juggle daily demands. The music therapist should focus more on solutions than problems, as everyone is an expert in their own reality, and all experiences should be acknowledged. All families have their own core values and beliefs and their own unique set of challenges.

The music therapist can act as a role model for healthy and clear interaction and communication, but also needs to be constantly aware of not overshadowing the parent by forming a strong alliance with the child or conversely forming a strong alliance with the parent and overshadowing the child. Imbalances in alliance must be avoided. The music therapist also acts as a facilitator of parent–child interaction and attachment, and here it is often an advantage to be able to switch modality and go to the music, where it is possible to take on several roles simultaneously. The music therapist is therefore active in musical activities. As the approach is family-centred, the whole family is involved and there is a strong focus on orienting and adapting to the family's dynamics, patterns, and emotional needs. The music therapist also adapts to the family's musical skills and focuses on being welcoming, inviting, playful and flexible. The music therapist might help facilitate the use of instruments and may also explain how music can be used intentionally but this is not essential. The music therapist is careful to refer according to current ethical guidelines and chooses activities that are appropriate to their own musical and therapeutic skills [38].

Music has a specific role, which links strongly to underlying psychology around parent–child interaction and relationship [38]. The family's musical interaction can be understood as a reflection or mirror of the family's general communication patterns, and therefore the family's interaction can be strengthened and vitalised through music. Musical interaction between family members facilitates dynamic and varied social interaction and, depending on the need, the focus can be on the training of social skills in the form of, for example, mutual turn-taking or exploration of different patterns and roles. Music motivates engagement and involvement through play because music facilitates play and the inherent communicative functions of play [38]. Through musical play, interaction occurs naturally and spontaneously, and in music parents and children can experience intimate encounters and try out new patterns through shared timing, rhythm, pulse, melody, and pitch. Music enables the sharing of feelings and thoughts, and can basically act as a common third, can help reminisce, explore identity, and sometimes bring aesthetic and vitalising experiences [39].

In terms of activities, the primary focus is on improvisation, exploring non-verbal emotional communication and allowing the family to explore and experience their own and others' family patterns, family traits, and family identity through music. The aim is to set up opportunities for family members to become accessible to each other and to share meaningful moments. Activities are group-based, with the music therapist inviting the use of different instruments and different ways of using the instruments. The music therapist may also use other music therapy techniques including song-writing and receptive methods, but these are not considered as primary activities. Mainly, the music therapist should be guided by what the families themselves want and adapt the activities flexibly and accordingly [38].

### Community music intervention

The unique and essential goal of the intervention is to enable positive shared musical experiences and to enable musical expression. It is about exploring and enhancing musical creativity and musical abilities, and this is unique [40]. Of course, it must also be a safe place for the family, so that individual self-esteem can be strengthened. For some families it may be about discovering genres or different preferences within the family, while for others it may be about forming new friendships across families or deepening existing relationships, but this is not essential.

The approach to music with families is mainly resource-oriented, with a focus on flexibility, diversity, and facilitation. There is a parallel focus on process and product, and much of the approach is about listening and adapting to families' individual skills social as well as musical and allowing space for diversity across. It is about seeing opportunities rather than constraints and creating a safe space that is accessible to all and where everyone makes space for each other [46]. At the beginning of the course, the focus is on getting to know each other and creating a safe space.

The role of the facilitator is to empower and make music accessible to families. Although the activities are based on the families' own social and personal skills and preferences, it is the facilitator who initiates and leads the activities to enable participation by all families and family members—possibly in turn [41]. It is about being welcoming, playful and flexible and helping families to get started using instruments or voice and including families and letting them be co-creators. The facilitator is careful to choose activities that suit the families and their own personal musical level and individual pedagogical considerations. Sometimes it makes sense to motivate families through their own musical skills, and other times it can have the opposite effect. It is the facilitator's role to keep an eye on this balance [42].

Everyone has the right and ability to create and enjoy their own music. The role of music is to enable shared aesthetic and revitalizing experiences and expressions. Music can be understood as a common third, and as something that enables social interaction [41, 42]. For some families it may be about exploring identity and evoking memories, but this is not essential.

The content and activities of the sessions consist of structured activities that facilitate positive musical experiences and expression. Repetition and a sense of practicing something together, collaborating on a specific product is unique and essential. The facilitator guides primarily group-based use of music, and other types of community music-focused activities such as singing games, circle songs, stomp elements, etc. may also be included but this is not essential. The facilitator has an eye on the difficulty of the activities also in relation to the start of the process and facilitating a safe space for everyone.

### Training facilitators

For it to make sense to compare and differentiate between the two active music interventions, according to Waltz [34] it is necessary to describe them in detail and train facilitators of the intervention thoroughly. The MUFASA group worked towards making the level of “essential and unique” obviously different while elements of both “essential” and especially “compatible” and “proscribed” are more alike. The facilitators were trained separately and their training including reading an elaborate intervention guide, reading relevant literature, and trying out the interventions in a role model teaching format where co-trainees and voluntary music therapy students roleplayed different types of family dyads with children in the age of 7–8. Based on short descriptions of the dyads, the facilitator-trainee planned a session with activities, tried it out in the role-model format and received feedback from peers as well as members from the MUFASA group. The group discussions were also used to clarify and edit elements of the written treatment guideline. The trainees only had access to one intervention guideline to avoid confusion about differences and similarities and to not be influenced by the other guideline.

### Concerts for families

The primary goal for family-concerts is to provide families with shared and meaningful experiences to strengthen family well-being. The criteria for which family concerts families should be offered to participate in are developed together with Musikkens Hus in Aalborg and Musikhuset in Aarhus, respectively, which also have many years of successful experience in offering activities for families with school children. Due to Covid19, it was not possible during the pilot study to choose or have families participate in family concerts. However, based on discussion with Musikkens Hus and Musikhuset the following criteria were chosen for the main study; concerts aimed at families in their description and/or title including both classical music and rhythmical music in both small and larger ensembles. The rationale behind these criteria is that included concerts should portray or represent regular possibilities in everyday life for families with children aged 7–9.

### Assessment of treatment fidelity

As mentioned, it is necessary to monitor whether the planned intervention is also the intervention delivered in the study [34]. Therefore, both facilitator as well as independent raters in the MUFASA project are to evaluate if it is possible to follow the intervention guide. Partly to know more about what is happening during the sessions so that we can understand the outcomes, but also for us to be able to validate the interventions. Below you can read the preliminary forms that we expect will be filled in partly after each session by the therapist and by independent raters who will watch random videos from the sessions. The treatment fidelity forms are strongly linked to the intervention guides and should be familiar to the facilitator.

### Power calculation and sample size

As no similar studies have been carried out previously, the basis for this calculation is sparse. There is no consensus on the minimal clinical difference in measures of parent-stress, child well-being, and parent–child interaction when participating in music activities. Overall, effect sizes appear to range from medium to large, e.g., between d = 0.57 and 1.60, and to be evident in similar studies where families participate in music and music therapy activities [43, 44].

The power calculation for the primary outcome measure APCI is based on a comparison between music therapy with vulnerable families and music therapy with more well-off families. For 32 randomised parent–child pairs, 90% power will be achieved for effect sizes between 0.57 and 1.6. Assuming drop-out does not exceed 10%, the aim is to recruit approximately 40 participants in total in addition to the pilot families.

### Outcomes measures

The study will use assessments by blinded clinicians as well as reports by parents/guardians. The music therapy activities will take place from 18 to 22 months and will be supported by an experienced family supervisor. The family concerts will take place 18–24 month, where the families' participation is closely followed by the project manager also through questionnaires. Measures of mental health and family interaction will be taken before and after participation in music and music therapy activities and subsequently one year after (months 18, 22 and 34) three times in total. The APCI is the primary outcome, and the PSS and the SDQ the secondary outcome measures. Besides these standardized questionnaires, the study will also collect data about family's everyday participation in other cultural or music activities and whether there is any shared music activates besides the activities offered in the MUFASA study.

Assessment of Parent Child Interaction (APCI) combines structure with flexibility and measures the actual interaction between parent and child. It is an observational and improvisational music therapy assessment tool with validity and reliability evidence [45, 46] that assesses the dyads of parent and child (5–12 years). The family sees the music therapist for 2 × 25 min with a week in between the two sessions, during which they do various exercises together and their interaction is videorecorded. The tool is thus based on video analysis and produces information about parent–child interaction and parenting skills including degree of mutual attunement, nonverbal communication, indication of attachment and degree of emotional parent response [47, 48].

Parental Stress Scale (PSS) is an internationally used questionnaire with evidence of validity and reliability [38] that assesses positive and negative aspects of parenting, including perceived stress, positive emotions, or experiences with parenting (including emotional gains, enrichment, and personal development), and negative aspects of parenting, such as guilt or lifestyle limitations. The positive and negative aspects of parenting are elucidated through 18 questions resulting in an overall parenting stress score [49].

The Strengths and Difficulties Questionnaire (SDQ) is a short questionnaire with evidence of validity and reliability [39] that assess children and young people's psychological well-being and functioning. It has been tested worldwide and translated into over 70 different languages and dialects. The SDQ has 25 questions and five additional questions asking the respondent (parents, when the child is under 11) to rate the duration of difficulties and their impact on everyday life, the latter being expressed in a strain score. The 25 questions have five questions on strengths and 20 questions on difficulties [50].

### Data management and storage

As the principal investigator and first author has no active task in assessing, rating, or facilitating intervention in connection with data collection, together with the second author they have shared responsibility to enter, code, anonymize, store and secure data using legal university software and storage with the aim of promoting data quality and accuracy. Furthermore, data monitoring is also handled by PI using closed excel sheets and SPSS docs. All participants are informed about legal GDPR storage of data including anonymization (code for finding individuals are available as long as the study runs and is then deleted) and rights and possibilities to cancel participation. Each participant gives individual consent to publication and different types of usage of video material for research analysis and training purposes.

### Statistical analysis

To examine the effect, repeated measures between groups (split-plot factorial) are used, where families are randomly assigned to participate in 4 different activities. Furthermore, adjusted linear mixed-effects models (LMEMs) are used to account for random effects in relation to participation in other cultural activities over the 3 years of the study. Measures are taken before music activities, shortly after and long after families have participated in music activities (see Fig. 1). To examine the relationship between parent-stress, child well-being and parent–child interaction and cultural participation, extended correlation analyses are also used, focusing on change over time and with time as a factor. A Mauchly test will be calculated for each variable to ensure the assumption of sphericity. Likewise, a Box's test will be performed to ensure covariance (equality of covariance). An alpha level of 0.05 is planned to be used for all statistical tests. If necessary, missing observations can be estimated and degrees of freedom should be adjusted downwards accordingly. Effect sizes are measured by calculating Cohen's d using the average of the standard deviations from pre- and post-measurements in both groups and conditions and adjusting for the correlation between the three measurements.

Different correlation measures will be used in this study to verify the degree of association between the APCI measures with that of the PSS and SDQ questionnaires in each of the different interventions, considering the different timepoints. These correlations are important because the constructs of each outcome measure are not the same, but they measure related constructs that may be associated in some way.

## Discussion

We aim to discuss results and clinical applicability of the findings looking at possible effect, possible patterns and correlations between intervention conditions and different types of families. We want to know if different formats of music experiences can strengthen different families in alternating ways, their interactions, relationships as well as individual measures of well-being and parental stress. As we are still in a post pandemic period, we anticipate that recruitment will still be challenging even if the need of families might be more evident than usual.

The study has limitations related to the fact that there are no other similar studies to compare with which weakens the validity of the power calculation. Furthermore, it is impossible to accurately evaluate, measure or control the impact of other parallel interventions or music activities that families are receiving or attending even though we collect this information. The two active interventions seem quite similar reading the intervention guide even if their unique and essential elements are different. However, experience from the pilot showcase evident differences when looking at video recordings of the session and this will be further discussed as well.

Findings of this study is aimed to be published in national and international peer-reviewed journals and through more public and accessible channels to reach any family member in a community. Findings will be relevant for other fields where music is applied for families, such as education, mental health, social work and for basic research on the study on music and families. If music is to be used as a resource for families in general, it is crucial to understand how different modalities can influence the family relationship, and how we might be able to strengthen interaction and emotional communication.

## Supplementary Information

**Additional file 1.** A checklist providing page numbers for each SPIRIT item adressed in this protocol.

**Additional file 2.** Documentation of extempt from ethical approval from regional Danish ethics committe - in Danish.

**Additional file 3.** Documentation of funding for the MUFASA research project - in Danish.

## Data Availability

Access to data related to the study protocol can be obtained by contacting the first author.

## Abbreviations

APCI: Assessment of parent child interaction (outcome measure)
GDPR: General data protection regulation
PPS: Parental stress scale (outcome measure)
MUFASA: Music, families and interaction (name of the study)
RCT: Randomised controlled study
SDQ: Strengths and difficulties questionnaire (outcome measure)
