# Supplementary material for: Music, families and interaction (MUFASA): a protocol article for an RCT study
Source: BMC Psychol. 2022 Nov 4;10:252. doi: 10.1186/s40359-022-00957-8 (PMC9636822; doi:10.1186/s40359-022-00957-8)
Supplement: Supplementary file 2 — Additional file 2. Documentation of extempt from ethical approval from regional Danish ethics committe - in Danish. [file 40359_2022_957_MOESM2_ESM.pdf]

**Fra:**	Videnskabsetisk Komité
**Til:**	Stine Lindahl Jacobsen
**Emne:**	SV: Forespørgsel på anmeldelsespligt
**Dato:**	19. september 2019 13:23:11

Kære Stine Lindahl Jacobsen

Du har ved mail af 19. september 2019 forespurgt Den Videnskabsetiske Komité for Region Nordjylland om anmeldelsespligt af dit planlagte projekt.

Komitélovens definition på et sundhedsvidenskabeligt forskningsprojekt omfatter behandling, undersøgelse, forebyggelse og rehabilitering som har til formål, at erhverve viden om sygdommes (somatiske, psykiatriske og ikke klinisk-psykologiske sygdomme og tilstande) opståen og om forebyggelse, diagnostik og behandling heraf.

På baggrund af de fremsendte oplysninger om, at det drejer sig om en undersøgelse omkring styrkelse af familiesamspil gennem aktive og observerende aktiviteter, - er det sekretariatets opfattelse, at projektet *ikke* er omfattet af komitélovens (LBK nr 1083 af 15/09/2017) definition på et sundhedsvidenskabeligt forskningsprojekt og derfor ikke skal anmeldes til og godkendes af komitéen, jf. komitélovens § 14, stk. 1, jf. § 2, nr. 1-3.

Projektet kan iværksættes uden yderligere tilbagemelding fra Den Videnskabsetiske Komité for Region Nordjylland.

Klagevejledning:
Afgørelsen kan, jf. komitélovens § 26, stk. 1, indbringes for National Videnskabsetisk Komité senest 30 dage efter, afgørelsen er modtaget. National Videnskabsetisk Komité kan, af hensyn til sikring af forsøgspersoners rettigheder, behandle elementer af projektet, som ikke er omfattet af selve klagen. Klagen samt alle sagens dokumenter sendes til: National Videnskabsetisk Komité – dketik@dketik.dk (sikker mail).

-
Vær opmærksom på at der kan være andre myndigheder, der skal godkende dit projekt. Komitéen kan ikke være behjælpelig med vejledning herom, men skal dog gøre opmærksom på, at der kan være krav om forudgående tilladelse fra Styrelsen for Patientsikkerhed, hvis du ønsker at anvende oplysninger fra patientjournaler til projektet – læs her for mere information.

Med venlig hilsen

Ulla Bay Hansen
Sekretær

SEKRETARIATET for DEN VIDENSKABSETISKE KOMITÉ for REGION NORDJYLLAND
Niels Bohrs Vej 30
9220 Aalborg Ø
Tlf. 97 64 84 40
vek@rn.dk
www.vek.rn.dk

Officiel post og post med digital signatur sendes til vek@rn.dk
Her kan du læse om dine rettigheder, når regionen behandler oplysninger om dig.

Fra: Stine Lindahl Jacobsen [mailto:slj@hum.aau.dk]
Sendt: 19. september 2019 12:43
Til: Videnskabsetisk Komité <vek@rn.dk>
Emne: Forespørgsel på anmeldelsespligt

Til rette vedkommende

Jeg er lektor og ansat ved Aalborg Universitet og har fra Det Obelske Familiefond fået bevilliget midler til at starte et forskningsprojekt med start 1.januar 2020 (dataindsamling efterår 2020). Jeg er i tvivl om, hvorvidt projektet er anmeldelsespligtigt, idet det ikke indeholder biologisk materiale eller sårbare patientgrupper. Forskellige sundhedsfokuserede aktiviteter og indsatser sammenlignes dog på tværs, og målet er at skabe ny viden samt efterprøve eksisterende viden. Nedenfor følger en kort beskrivelse af projektet:

Projekts formål at styrke sundt familiesamspil gennem aktive og observerende aktiviteter på tværs af musikaktiviteter, musikterapiaktiviteter og familie musikkoncerter. Der er fokus på forebyggelse og en ressource-fokuseret tilgang, hvor mental sundhed, familieinteraktion og tilknytning styrkes. Det planlægges at tilbyde aktiviteter til 40 frivillige familier med skolebørn i alderen 7-10 år i Aalborg og Aarhus kommune, hvor familierne inddeles tilfældigt i 4 forskellige grupper, der deltager i hhv. musikaktiviteter, musikterapiaktiviteter, familiemusikkoncerter samt en gruppe, hvor deres sædvanlige deltagelse i musik og kulturaktiviteter kortlægges. Alle familiers øvrige kultur-deltagelse undersøges gennem et uddybet spørgeskema, så aktiviteterne kan sammenlignes på tværs også i forhold til øvrig deltagelse i musik- og kulturaktiviteter. De 40 familier følges over en årrække, så også en eventuelt langtidseffekt af musik og kulturaktiviteterne til familier med skolebørn kan undersøges. Ud over kortlægning af musik og kulturdeltagelse indsamles der informationer løbende om familiemedlemmernes trivsel, familiesamspil og tilknytning. Informationerne indsamles gennem spørgeskemaer og observationsredskaber (herunder brug af video).

Jeg ser frem til at høre fra jer og indsender gerne en udførlig protokol, såfremt det er relevant.

Med venlig hilsen
Stine Lindahl Jacobsen

Stine Lindahl Jacobsen
Ph.d., Associate professor| Head of Music Therapy Programme
Department of Communication & Psychology | Aalborg Universitet|Musikkens Plads 1 | 9000 Aalborg,
Contact: slj@hum.aau.dk | (+45) 99409103
