# Supplementary material for: Music, families and interaction (MUFASA): a protocol article for an RCT study
Source: BMC Psychol. 2022 Nov 4;10:252. doi: 10.1186/s40359-022-00957-8 (PMC9636822; doi:10.1186/s40359-022-00957-8)
Supplement: Supplementary file 3 — Additional file 3. Documentation of funding for the MUFASA research project - in Danish. [file 40359_2022_957_MOESM3_ESM.pdf]

Institutet for Kommunikation og Psykologi, Aalborg Universitet
Musikkens Plads 1
9000 Aalborg
Att: Stine Jacobsen

DET OBELSKE FAMILIEFOND

Aalborg, den 09. maj 2019

Sagsnummer: 30485
Mental Sundhed i Familien. Musikoplevelser for familier med skolebørn

Kære Stine Jacobsen,

Det Obelske Familiefond har bevilget

kr. 2.096.220,00
til: **Mental Sundhed i Familien. Musikoplevelser for familier med skolebørn.**

Jeres kontaktperson i fondet er projektchef Peter Larsen (pl@obel.com).

Vi beder jer holde os løbende orienteret om projektets udvikling gennem hele bevillingsperioden.

Inden for den kommende måned skal I kontakte fondet med et forslag til en tidsplan for udbetalingsrater. Kontakt samt upload af statusrapporter og slutrapport skal ske via 'Min side'.

Læs om fondets krav til bevillingsmodtagere
her: http://obel.com/soeg-stoette/til-bevillingsmodtagere

Samarbejdet med Det Obelske Familiefond skal nævnes i forbindelse med eventuel presse eller anden offentlighed.

Med venlig hilsen
Det Obelske Familiefond

Michael Bjørn Nellemann
Direktør

Kastetvej 2
9000 Aalborg
Tel.: 98 12 73 00
dof@obel.com
CVR: 31869528
